# Diet patterns are associated with demographic factors and nutritional status in South Indian children

**DOI:** 10.1111/mcn.12046

**Published:** 2013-07-02

**Authors:** Sarah H. Kehoe, Ghattu V. Krishnaveni, Sargoor R. Veena, Aravinda M. Guntupalli, Barrie M. Margetts, Caroline H.D. Fall, Sian M. Robinson

**Affiliations:** ^1^ Medical Research Council Lifecourse Epidemiology Unit University of Southampton Southampton UK; ^2^ Medical Research Council Holdsworth Memorial Hospital Mysore India; ^3^ Faculty of Medicine Primary Care and Population Sciences University of Southampton Southampton UK

**Keywords:** child, chronic disease, diet pattern, India, nutritional status

## Abstract

The burden of non‐communicable chronic disease (NCD) in India is increasing. Diet and body composition ‘track’ from childhood into adult life and contribute to the development of risk factors for NCD. Little is known about the diet patterns of Indian children. We aimed to identify diet patterns and study associations with body composition and socio‐demographic factors in the Mysore Parthenon Study cohort. We collected anthropometric and demographic data from children aged 9.5 years (n = 538). We also administered a food frequency questionnaire and measured fasting blood concentrations of folate and vitamin B12. Using principal component analysis, we identified two diet patterns. The ‘snack and fruit’ pattern was characterised by frequent intakes of snacks, fruit, sweetened drinks, rice and meat dishes and leavened breads. The ‘lacto‐vegetarian’ pattern was characterised by frequent intakes of finger millet, vegetarian rice dishes, yoghurt, vegetable dishes and infrequent meat consumption. Adherence to the ‘snack and fruit’ pattern was associated with season, being Muslim and urban dwelling. Adherence to the lacto‐vegetarian pattern was associated with being Hindu, rural dwelling and a lower maternal body mass index. The ‘snack and fruit’ pattern was negatively associated with the child's adiposity. The lacto‐vegetarian pattern was positively associated with blood folate concentration and negatively with vitamin B12 concentration. This study provides new information on correlates of diet patterns in Indian children and how diet relates to nutritional status. Follow‐up of these children will be important to determine the role of these differences in diet in the development of risk factors for NCD including body composition.

## Introduction

The World Health Organisation (WHO) has estimated that 53% of all deaths in India in 2008 were attributable to non‐communicable conditions such as cardiovascular disease and type 2 diabetes (World Health Organisation 2011). It is predicted that by 2030, such conditions will account for 75% of deaths (Patel *et al*. 2011). Since the 1970s, there has been a reduction in intakes of wholegrain cereals, pulses, fruits and vegetables in India while intakes of meat products, refined grains and salt have increased (Popkin 2002; Misra *et al*. 2011; Popkin *et al*. 2012). In parallel with these dietary changes, there is evidence of increased prevalence of child and adolescent overweight. It has been estimated that in 2011, the prevalence of childhood overweight was 15 million and that of abdominal obesity was 4 million (Gupta *et al*. 2012). Data from large cross‐sectional surveys in Delhi have shown that there was an increase in adolescent obesity from 9.8% in 2006 to 11.7% in 2009 (Pandey *et al*. 2009). In Kerala, two cross‐sectional surveys of more than 20 000 children found that the percentage of overweight children increased from 4.94% to 6.57% between 2003 and 2005 (Raj *et al*. 2007). Recent data suggest that prevalence of adolescent overweight is almost 20% in urban areas (Goyal *et al*. 2010; Jain *et al*. 2010).

Diet and body composition are important contributory factors to chronic disease risk in adult life (Shetty 2002; Misra *et al*. 2011). Since evidence suggests that dietary patterns ‘track’ from childhood to adulthood (Dunn *et al*. 2000; Mikkila *et al*. 2005), it is important to study diet patterns during childhood as this may provide an opportunity to intervene and prevent chronic disease. However, little is currently known about the dietary patterns of children in India. Studies in European children have looked at the associations between diet patterns and body composition and found that ‘snacking’ and ‘energy dense’ patterns are associated with overweight (Lioret *et al*. 2008) and higher fat mass (Johnson *et al*. 2008). South Asian children tend to have a higher body fat percentage compared with Western children (Yajnik *et al*. 2003; Krishnaveni *et al*. 2005), but to our knowledge diet patterns and their relationships with body composition have not been studied among South Asian children. Despite the rapid urbanisation currently occurring in South Asia (United Nations Department of Economic and Social Affairs 2011), diet patterns are likely to differ from those of Western children.

The three aims of this study were: (1) to describe diet patterns of children living in and around the city of Mysore, located in central South India, using a principal component analysis (PCA) of food frequency data; (2) to examine demographic variables as correlates of diet patterns; and (3) to examine diet patterns as correlates of body composition and micronutrient status (folate and vitamin B12).

## Key messages

- There are few data on the dietary patterns of Indian children. Given that diet in childhood may affect chronic disease risk, we investigated the diet patterns of 9.5 year old South Indian children.
- Two diet patterns were identified: ‘snack and fruit’ and ‘lacto‐vegetarian’. Both patterns were associated with markers of nutritional status.
- Knowledge of these patterns may be useful for designing targeted interventions to improve nutritional status and for developing food based dietary guidelines in this population.

## Method

### Participants and Setting

Children were recruited from the Mysore Parthenon study, a birth cohort set up to investigate the long‐term cardiovascular risk outcomes associated with maternal gestational diabetes and body composition of the infant at birth. Details of the cohort have been published elsewhere (Hill *et al*. 2005). In brief, between June 1997 and August 1998, pregnant women attending the antenatal clinic of Holdsworth Memorial Hospital, living in the city of Mysore and surrounding rural areas were recruited to the study if they fulfilled the following criteria: non‐diabetic prior to pregnancy; <32 weeks gestation at time of recruitment; planning to deliver at Holdsworth Memorial Hospital. A total of 1233 women were eligible for the study, 830 (67%) agreed to participate. Babies were included in the study if they were singletons and had no major congenital anomalies; 663 of 674 babies met the inclusion criteria. Of the 663, 41 were born to women with gestational diabetes. In 2007, 539 (81%) children attended for follow‐up at 9.5 years (56 refused, 8 were not traced, 26 moved away, 25 died and nine were withdrawn from the study for medical reasons) Dietary data were available for 538 children. This study was conducted according to the guidelines laid down in the Declaration of Helsinki and all procedures involving human subjects were approved by the Holdsworth Memorial Hospital Ethics Committee.

### Procedure

Data on maternal parity and religion were collected during pregnancy. Parental education, occupation, socio‐economic status and family type were recorded at 9.5 years. In terms of education, parents were categorised as having completed: fewer than 10 years education (below secondary level); 10 years education (secondary level); or more than 10 years education (above secondary level). Occupations were classified as follows: professional, e.g. university teacher, chemist, lawyer; skilled, e.g. goldsmith, carpenter, police constable; unskilled, e.g. labourer, vegetable seller. Socio‐economic status data were collected using the Standard of Living questionnaire from the Indian National Family Health Survey (International Institute for Population Sciences (IIPS) 2000). The respondent was the child's parent or guardian. Children were classified as living in an urban or rural area based on their address at 9.5 years. We defined towns with a population greater than 100 000 as urban areas based on Indian Government census data (Government of India 2012). We used data collected at the time of pregnancy to determine the proportion of the cohort that had migrated from rural to urban areas or vice versa. As close as possible to the age of 9.5 years, children were asked to visit the research centre at the Holdsworth Memorial Hospital, accompanied by their parents. A food frequency questionnaire (FFQ) was administered by one of three trained nutritionists. Child and maternal anthropometry, and collection of blood samples, were carried out on the same day.

### Measurement of Blood Nutrient Concentrations

We chose to measure plasma folate and vitamin B12 concentrations in the children as there has recently been significant interest in the status of these nutrients and later risk of chronic disease (Yajnik *et al*. 2006, 2008; Christian & Stewart 2010). In addition, a large proportion of the Indian population are vegetarian and there is some evidence of vitamin B12 deficiency in certain settings (Yajnik *et al*. 2006; Pathak *et al*. 2007; Krishnaveni *et al*. 2009). Parents and children were asked in a neutral manner whether the child had consumed any food or drink other than water in the 12 h prior to the appointment, if the child was not fasted an alternative appointment was made for another day. Fasting venous blood samples (15 mL) were collected. Plasma was separated and stored at −80°C prior to analysis. Plasma concentrations of folate and vitamin B12 were determined by microbiological assay (Kelleher *et al*. 1987; Horne & Patterson 1988) at the Diabetes Unit, KEM Hospital Research Centre, Pune, India. Intra‐ and inter‐assay coefficients of variation (CV) were <8% for both assays.

### Dietary Assessment

A trained nutritionist interviewed the child and a parent or guardian (usually the mother) in order to collect dietary data; both child and adult provided information. A 136‐item FFQ (see http://onlinelibrary.wiley.com/doi/10.1111/mcn.12046/suppinfo) was developed based on responses to 24 h recalls administered to children in the cohort at 8 years. All food and drinks reported were listed and local nutritionists added any foods that had not been reported during the recalls but were thought to be consumed by the study population. In order to help respondents to conceptualise the child's diet (Cade *et al*. 2002), the FFQ foods were then divided into 15 categories (beverages; fruit; dried fruit and nuts; rice foods; wheat foods; finger millet (ragi) foods; cooked vegetable dishes; salad; meat/poultry and eggs; jam/chutney; sugar added to foods; savoury snacks; sweet snacks; fast food; milk/ milk products). The reference time period for the FFQ was a typical month and the response categories were daily, weekly or monthly with participants stating on how many occasions per day, week or month they consumed the food item. To differentiate between children who were consuming six varieties of seasonal fruit all year round and those who were only consuming them when in season, there were two items on the FFQ for each fruit corresponding to each scenario. When it was reported that fruit was consumed seasonally, a correction factor was applied to the frequency to take account of the number of months of the year that the fruit was available, e.g. mangos are generally available for a quarter (3 months) of the year so the frequency was multiplied by 0.25. The season in which the FFQ was administered was recorded. The Indian Government Meteorological Department season classifications were used: Winter, January and February; Pre‐monsoon, March, April, May; Monsoon, June, July, August and September; Post‐monsoon, October, November and December (available at: http://www.imd.gov.in/doc/termglossary.pdf).

### Anthropometry and Bio‐impedance Measurements

Height was measured to the nearest 0.1 cm using a microtoise wall‐mounted stadiometer (CMS Instruments, London, UK). Weight was measured to the nearest 100 g using an electronic digital weighing scale (Salter, Kent, UK). Body mass index (BMI) was calculated as weight divided by the square of height (kg m^−2^). Weight and BMI were compared with WHO international child growth standards (World Health Organisation 2007). Head, chest, mid‐upper arm and abdominal circumferences were all measured to the nearest mm using anthropometric tape. Subscapular and triceps skinfold measurements were made in triplicate using Harpenden callipers (CMS Instruments) and the mean was used in the analyses. All measurements were made on the left side of the body. There were five measurers and mean intra‐observer CVs were 1.9% for triceps and 4.0% for subscapular skinfolds; mean inter‐observer CVs were 6.8% for triceps and 7.7% for subscapular skinfolds.

Whole body impedance at 50 kHz was measured using a Quadscan 4000 analyser according to the manufacturer's instructions (Bodystat, Isle of Man, British Isles). Impedance (Ω) at 50 kHz and percentage body fat, based on the pre‐programmed Bodystat equation for children, were recorded. The CV for impedance measurements was <1%.

### Statistical Analysis

#### Diet patterns analysis

The 136 foods on the FFQ were condensed to 52 food groups based on nutrient content and typical use, for example puffed rice and rice flakes were grouped as ‘processed rice foods’ and cabbage palya (cooked vegetable preparation served dry), green leafy vegetable palya and green leafy vegetable curry were grouped as ‘green leafy vegetable dishes’. PCA without rotation was used to identify the children's diet patterns. PCA is a statistical technique that has been widely used in diet patterns research (Hu 2002; Newby & Tucker 2004). It identifies foods that are consumed together and produces new variables (components) that are independent linear combinations of the dietary variables accounting for maximum variance (Joliffe & Morgan 1992). Each component can represent a particular diet pattern. Pattern scores were calculated for each child based on the weekly frequency of consumption of items from the food group and the coefficient for that food group. Weekly frequencies were calculated by multiplying daily intakes by 7 or by dividing monthly intakes by 4. These values were summed for all 52 food groups to provide scores that represent the child's adherence to a particular pattern. Pattern *z*‐scores were then calculated with a mean (SD) of 0 (1). The number of diet patterns described in the results section was based on the identification of a break in the scree plot, the component eigenvalue being >2 and interpretability of the pattern for this population. Food groups with coefficients >|0.2| were considered to be discriminatory.

#### Correlate and outcome variables

Variables that were not normally distributed were transformed by taking natural logarithms. Differences in mean pattern scores between boys and girls were examined using *t*‐tests. Analyses of variance (ANOVAs) and correlation coefficients were used to assess univariate associations between predictor variables and pattern scores. Correlate variables were season of the year that the FFQ was administered, socio‐demographic characteristics and maternal BMI. All variables significantly associated with pattern score in the univariate analyses were entered into multivariate regression models to identify independent correlates of pattern scores. Based on the findings, *post hoc* two‐factor ANOVAs were used to test for interaction. The associations between pattern scores and nutritional status of the child including height, BMI, folate and vitamin B12 concentrations, and body composition were assessed using the Wilcoxon rank‐sum test for trend across all four quarters of pattern score.

All data were analysed using Stata version 11 (Stata Corporation, College Station, TX, USA).

## Results

Table 1 shows descriptive and anthropometric data relating to the 538 children (254 male) studied, together with parental and family characteristics. Children were on average 2 SD lighter and had a median BMI approximately 1 SD lower than the WHO international reference population (World Health Organisation 2007). Almost two‐thirds of mothers and fathers had at least 10 years education, half of the mothers had a BMI >25 kg m^−2^. The proportion of families that were Muslim was considerably greater than the Indian national survey figure of 12.5% (International Institute for Population Sciences (IIPS) and Macro Interntional 2007). The proportion of nuclear families was almost identical to the survey figure of 60.5%. Three quarters of the families lived in urban areas. The majority (91%) of children were living in the same setting as they had been since birth. Of the 50 children whose status had changed, 32 had moved from rural to urban areas and 18 had moved from urban to rural.

**Table 1:** Participant and family characteristics

	Variable		*n*	%	Mean	SD
Child	Age (years)		538		9.4	0.1
	Height (cm)		538		130.7	5.7
	Weight (kg)*		538		24.4	(22.2–27.4)
	Body mass index (kg m^−2^)*		538		14.3	(13.4–15.6)
	Mid‐upper arm circumference (cm)*		538		17.7	(16.9–19.1)
	Head circumference (cm)		538		50.6	1.4
	Subscapular skinfold (mm)*		537		7.2	(5.7–9.2)
	Triceps skinfold (mm)*		538		9.4	(7.6–12.0)
	Fat mass (kg)*		538		6.7	(5.2–8.4)
	Fat percentage		538		27.5	(6.6)
	Plasma vitamin B12 (pmol L^−1^)*		527		312	(249–407)
	Plasma folate (nmol L^−1^)*		527		24.0	(18.0–35.0)
Mother	Education (years)	<10	199	37.0		
		10	167	31.0		
		>10	172	32.0		
	Parity^†^	0	275	51.1		
		1	175	32.5		
		>1	88	16.4		
	Body mass index (kg m^−2^)	<18.5	30	5.8		
		18.5–25.0	224	43.4		
		25.1–30.0	184	35.7		
		>30.0	78	15.1		
Father	Education (years)	<10	206	38.3		
		10	114	21.2		
		>10	218	40.5		
Family	Religion	Hindu	306	56.9		
		Muslim	190	35.3		
		Other	42	7.8		
	Dwelling	Rural	136	25.3		
		Urban	402	74.7		
	Family type	Nuclear	324	60.3		
		Joint	213	39.7		
	Occupation^‡^	Unemployed/Unskilled	27	5.0		
		Semi‐skilled	88	16.4		
		Skilled and clerical	357	66.4		
		Semi‐professional/Professional	66	12.3		
		Standard of Living (SLI score)^§^	538		36.3	8.2

*Values are median (inter‐quartile range). ^†^Parity refers to the time of recruitment into the cohort study i.e. the time the participant in the cohort study was conceived. ^‡^Refers to occupation of the head of the household. ^§^Standard of Living Index used in the National Family Health Survey, India.

### Diet Patterns

We carried out separate PCA for girls and boys and found minimal differences in the patterns (data not shown) therefore, the data presented relate to the sexes combined. Based on the scree plot and eigenvalues, we examined the first three components to assess interpretability. The first component, which explained 9.1% of the variance, was named ‘snack and fruit’ and was characterised by high intakes of snacks, fresh fruit, sweetened drinks, rice and meat dishes (biryani), noodles and leavened breads. The second component explained 7.5% of the variance and was named the ‘lacto‐vegetarian’ diet as it was characterised by high intakes of finger millet (ragi), traditional rice dishes, yoghurt (curd), vegetable dishes, sugar added to foods and a low frequency of meat consumption. The third component explained less variance (4.1%). Its key characteristics were high intakes of malt‐based hot drinks and low intakes of tea and coffee but there was no clear pattern in the remaining food groups (data not shown). It was not considered to provide a meaningful or interpretable pattern of foods in the context of chronic disease risk and was not included in any further analyses. Table 2 shows the coefficients for each of the 52 food groups relating to the first and second components of the PCA. Consumption frequencies of the discriminating foods for each pattern (highlighted in bold in Table 2) for children with scores in the top and bottom quarters of the distributions are shown in Table 3. For the majority of these foods, there was a two‐ to ninefold difference in median frequency of intake between those in the lowest and highest quarters. The foods with the greatest differences for the snack and fruit pattern were sweetened drinks and fruit, while for the lacto‐vegetarian pattern they were finger millet (ragi) and yoghurt (curd).

**Table 2:** Table of food groups used in the principal component analysis of food frequency data with a description of the food group and the coefficients for each pattern identified

Food type	Food group	Description of food group	Coefficients*	
			S&F	LV
Beverages	Tea and coffee		−0.05	0.14
	Milk	Fresh milk	−0.01	−0.02
	Hot milky drinks	Drinks made with hot milk and processed powder products (brands include: ‘Horlicks’, ‘Complan’, ‘Boost’, ‘Bournvita’)	0.01	0.11
	Fruit juice	Fresh fruit juice	0.13	0.01
	Fruit‐based drinks	Processed fruit drinks containing added sugar (brands include: ‘Frooty’, ‘Maza’)	0.17	−0.05
	Sweetened drinks	Carbonated drinks; drinks with added flavouring and sugar (brands include: ‘Pepsi’, ‘Sprite’)	**0.23**	0.08
Fruit	Banana		0.10	0.12
	Apple		**0.21**	0.03
	‘Snack’ fruit**^†^**	Guava, pomegranate, grapes, sweet lime (*Citrus Limetta*), orange	**0.25**	0.15
	Other fruit	Sapota (*Manilkara zapota)*; Watermelon; Papaya; Jack‐fruit (*Artocarpus heterophyllus*); Mango	**0.20**	0.07
	Dry fruit and nut	Dates, raisins, cashew nuts, pistachios, almonds	0.13	0.00
Rice	Rice	Plain steamed rice	0.01	0.07
	Rice with Dahl	Rice with lentils	0.12	−0.14
	Traditional rice	Rice flavoured with tamarind, rice flavoured with lemon	0.07	**0.22**
	Oily rice	Rice fried with oil or ghee	**0.20**	−0.03
	Rice with vegetables	Rice with vegetables and oil	0.02	0.16
	Rice with yoghurt	Rice with yoghurt (milk curd)	0.07	**0.26**
	Processed rice	Puffed Rice, Rice Flakes	0.12	**0.21**
Fermented rice foods	Idly	Steamed patty made from fermented rice and lentils	0.02	0.03
	Rice dosas	Pancake made from fermented rice and lentil	0.01	**0.20**
	Masala dosa	Pancake made from fermented rice and lentils stuffed with potato and spices	0.05	0.08
Bread	Unleavened breads	Chapathi, Parata *(wheat based unleavened breads)*	0.11	−0.16
	Unleavened oily bread	Poori *(fried wheat based unleavened bread)*	0.12	0.12
	Leavened breads	Bun, sliced bread	**0.20**	0.02
Other cereal based foods	Branded noodles	Wheat noodles (brands include ‘Maggi’)	**0.21**	−0.15
	Finger millet^‡^	Finger millet bread; finger millet porridge	0.01	**0.27**
	Wheat dishes	Semolina, wheat vermicelli	0.06	0.07
Cooked vegetable dishes	Dry cooked vegetables dishes	Pea, French bean, eggplant, carrot or beetroot cooked dry with spices	0.07	**0.23**
	Cooked GLV dishes	GLV cooked dry with spices, green leafy vegetable curry	0.01	0.06
	Cooked GLV dishes with lentils	GLV and lentil curry	0.02	0.19
	Whole legume curries	Curries made from whole (non‐split) lentils and other legumes	0.02	0.15
	Tomato curry	Curry made with tomato and onion	−0.02	0.18
	Dry potato dishes	Potato cooked with spices	0.09	0.00
Salad	Salad	Cucumber, tomato, onion, carrot, radish, beans, cabbage, beetroot	0.19	0.11
Meat/fish/egg dishes	Rice with poultry, meat or egg	Chicken, mutton or egg biryani (rice dish cooked with oil)	**0.23**	−0.17
	Fish	Fish, fried or curry	0.10	−0.12
	Chicken	Chicken, fried or curry	0.11	**−0.21**
	Mutton	Mutton, whole pieces or minced	0.16	**−0.26**
	Boiled eggs		0.09	−0.02
	Fried eggs	Fried egg, omelette	0.15	−0.19
Snacks	Cereal‐based snacks	Snacks made with wheat or rice egg samosa	**0.28**	0.07
	Legume‐based snacks	Snacks made with split or whole legumes egg pakora	**0.24**	−0.09
	Chips	Deep fried potato or plantain slices	0.19	−0.01
	Cakes	Plain cakes, cream cakes	0.19	−0.04
	Biscuits	Salted biscuit, sweet biscuit, cream biscuit	0.12	0.02
Sugary food/Sweets	Jam	Jam	0.17	−0.04
	Honey	Honey	0.11	0.04
	Added sugar	Sugar added to any food or added to fruit juice by the child/parent	0.01	**0.21**
	Confectionery	Chocolate bar, toffee, candy, ice cream, ice lolly	0.17	−0.12
	Home‐made sweets	Sweets, sweet vermicelli	**0.20**	−0.03
Dairy	Yoghurt	Yoghurt (milk curd), raita (*yoghurt and raw vegetables*), buttermilk	0.07	**0.24**
	Butter and ghee	Butter, ghee (*clarified butter*)	0.11	0.17
Chutney	Chutney	Chutney, pickle	−0.02	0.07
	**Total variance explained (%)**		**9.1**	**7.5**

GLV, green leafy vegetable; LV, lacto‐vegetarian pattern; S&F, snack and fruit pattern. *Values in bold are >|0.20|, ^†^Snack fruit is defined as fruit that is eaten in small quantities. ^‡^Finger millet (*Eleusine coracana*) is a cereal grown in arid climates.

**Table 3:** Monthly frequency of consumption of discriminating foods for each pattern by children whose pattern scores fall in the lowest and upper most quarters of the distribution*

	Lowest quarter (*n* = 135)	Highest quarter (*n* = 134)
**Snack and fruit pattern**		
Sweetened drinks	1.0 (0.0–3.0)	8.0 (4.0–16.0)
Apple	2.0 (0.0–4.0)	4.0 (2.0–14.0)
Snack fruit	6.0 (3.5–10.5)	25.5 (14.0–37.0)
Other fruit	8.0 (5.0–14.0)	20.0 (11.0–28.0)
Oily rice	1.0 (0.0–2.0)	4.0 (4.0–8.0)
Leavened breads	4.0 (1.0–6.0)	8.0 (7.0–16.0)
Rice with poultry, meat or egg	0.0 (0.0–0.0)	3.0 (1.0–5.0)
Cereal‐based snacks	13.0 (9.0–18.0)	40.0 (30.0–52.0)
Legume‐based snacks	5.0 (3.0–8.0)	16.0 (10.0–21.0)
Home‐made sweets	1.0 (0.0–2.0)	6.0 (2.0–12.0)
Branded noodles	0.0 (0.0–2.0)	4.0 (1.0–8.0)
**Lacto‐vegetarian pattern**		
Traditional rice	4.0 (0.0–7.0)	8.0 (6.0–8.0)
Rice with yoghurt	3.0 (0.0–4.0)	28.0 (8.0–28.0)
Fermented rice pancake	5.0 (4.0–8.0)	8.0 (8.0–12.0)
Processed rice	4.0 (0.0–8.0)	8.0 (4.0–12.0)
Finger millet	0.0 (0.0–1.0)	9.5 (3.0–28.0)
Cooked vegetable dishes	4.0 (1.0–8.0)	10.0 (6.0–15.0)
Chicken	4.0 (4.0–8.0)	2.0 (0.0–4.0)
Mutton	10.0 (7.0–15.0)	2.0 (0.0–4.0)
Added sugar	28.0 (16.0–36.0)	56.0 (56.0–64.0)
Yoghurt	0.0 (0.0–4.0)	9.0 (4.0–28.0)

*Values are median (inter‐quartile range). The Wilcoxon rank‐sum test showed a trend across quarters for all foods (*P* < 0.001).

### Associations between season, demographic characteristics and diet patterns

#### Snack and Fruit Pattern

Univariate analyses showed that the snack and fruit pattern was predicted by season of interview with the pre‐monsoon and monsoon seasons being associated with higher scores (Table 4). Muslim religion, living in an urban area, the head of the household having an unskilled occupation, and being part of a nuclear family were all significantly associated with scores on this pattern. The multivariate analysis suggested that season, religion and dwelling were all significant independent correlates of scores (Table 5). Season was the strongest independent correlate and explained half (13%) of the variance in pattern scores. The association between family type and pattern score was of borderline significance.

**Table 4:** Diet pattern *z*‐scores by child, parent and family characteristics

	*Variable*	*n*		Snack and fruit pattern		Lacto‐vegetarian pattern	
				Score*	*P* ^†^	Score*	*P* ^†^
Child	Gender	254	Male	0.06 (1.0)	0.176	−0.02 (1.0)	0.659
		284	Female	−0.05 (0.9)		0.02 (0.9)	
Mother	Education (years)	199	<10	−0.09 (1.1)	0.106	0.05 (0.9)	0.661
		167	10	0.13 (0.9)		−0.02 (0.9)	
		172	>10	−0.02 (0.9)		−0.04 (0.9)	
	Parity*	275	0	−0.04 (1.0)	0.151	−0.02 (0.9)	0.304
		175	1	−0.03 (0.9)		0.13 (0.9)	
		88	>1	0.19 (1.1)		−0.20 (1.1)	
	Body mass index (kg m^−2^)	30	<18.5	−0.09 (1.1)	0.194	0.36 (0.9)	<0.001
		224	18.5–25.0	−0.04 (0.9)		0.13 (0.9)	
		262	>25.0	0.06 (0.9)		−0.13 (0.9)	
Father	Education (years)	206	<10	0.07 (1.0)	0.365	−0.15 (1.0)	0.012
		114	10	−0.08 (0.9)		0.02 (0.9)	
		218	>10	−0.02 (0.9)		0.13 (0.9)	
Family	Religion	306	Hindu	−0.26 (0.9)	<0.001	0.59 (0.7)	<0.001
		190	Muslim	0.43 (0.9)		−0.93 (0.7)	
		42	Other	−0.06 (0.9)		−0.10 (0.7)	
	Dwelling	136	Rural	−0.31 (1.1)	<0.001	0.49 (0.8)	<0.001
		402	Urban	0.10 (0.9)		−0.17 (0.9)	
	Family type	324	Nuclear	0.09 (0.9)	0.009	0.03 (0.9)	0.381
		213	Joint	−0.14 (0.9)		−0.05 (0.9)	
	Occupation^‡^	115	Unskilled	0.22 (0.9)	0.013	−0.17 (1.0)	0.052
		357	Skilled	−0.09 (1.0)		0.02 (0.9)	
		66	Professional	0.08 (0.9)		0.19 (1.0)	
	Standard of living (SLI score)	135	1^st^ quarter	−0.02 (1.1)	0.649	−0.19 (1.0)	0.079
		134	2^nd^ quarter	0.05 (0.8)		−0.01 (0.9)	
		134	3^rd^ quarter	0.02 (1.1)		0.12 (0.9)	
		135	4^th^ quarter	−0.05 (0.9)		0.09 (0.9)	
Season		99	Winter	−0.61	<0.001	−0.12	0.005
		114	Pre‐monsoon	0.50		0.28	
		203	Monsoon	0.20		−0.10	
		121	Post‐monsoon	−0.31		0.00	

*Mean (SD) pattern *z*‐score, ^†^All analyses are univariate models, *P* values are based on t‐test or analysis of variance statistics except for Maternal body mass index and SLI where *P* values are based on correlation coefficients. ^‡^Refers to occupation of the head of the household. Unskilled category comprises unemployed, unskilled and semi‐skilled individuals.

**Table 5:** Multivariate regression analysis of variables associated with pattern scores

Snack and fruit pattern*		Beta	Confidence interval	*P*
Religion	Muslim	0.59	(0.42, 0.76)	<0.001
(Reference = Hindu)	Other	0.13	(−0.16, 0.41)	0.374
Dwelling	Urban	0.20	(0.02, 0.38)	0.026
(Reference = Rural)				
Family Type	Joint	−0.13	(−0.28, 0.02)	0.096
(Reference = Nuclear)				
Occupation	Skilled	−0.03	(−0.22, 0.15)	0.721
(Reference = unskilled)	Professional	0.09	(−0.17, 0.36)	0.491
Season	Pre‐monsoon	1.06	(0.83, 1.30)	<0.001
(Reference = winter)	Monsoon	0.72	(0.51, 0.93)	<0.001
	Post Monsoon	0.28	(0.05, 0.51)	0.017
Lacto‐vegetarian pattern^†^				
Maternal body mass index (kg m^−2^)		−0.02	(−0.03, −0.00)	0.021
Parity	1	0.08	(−0.06, 0.21)	0.254
(Reference = null)	>1	0.04	(−0.13, 0.21)	0.671
Father's education	10 years	0.06	(−0.10, 0.22)	0.459
(Reference ≤ 10 years)	>10 years	−0.04	(−0.18, 0.10)	0.575
Religion	Muslim	−1.48	(−1.62, −1.34)	<0.001
(Reference = Hindu)	Other	−0.63	(−0.86, −0.39)	<0.001
Dwelling	Urban	−0.17	(−0.31, −0.00)	0.023
(Reference = Rural)				
Season	Pre‐monsoon	0.42	(0.22, 0.64)	<0.001
(Reference = Winter)	Monsoon	0.17	(0.00, 0.34)	0.053
	Post‐monsoon	0.10	(−0.08, 0.29)	0.270

*Adjusted r square = 0.26. ^†^Adjusted r square = 0.54.

#### Lacto‐vegetarian Pattern

Season of FFQ administration was associated with the lacto‐vegetarian pattern, with those interviewed during the pre‐monsoon season having higher scores (Table 4). Maternal BMI was negatively associated with pattern scores while higher scores were predicted by being of Hindu religion, rural dwelling, greater duration of paternal education and skilled occupation of the head of household. In the multivariate model (Table 5), religion was the strongest correlate of pattern scores with maternal BMI, dwelling and season all being significant independent correlates. However, for this pattern season accounted for only 1% of the variance explained by the model. Religion alone accounted for 16% of the variance.

There was an apparent paradox whereby the children with higher scores on the lacto‐vegetarian pattern were more likely to live in rural areas but the occupation of the head of the household was more likely to be professional. We therefore explored the associations between standard of living and pattern scores in the urban and rural children separately and found that there was a positive association between standard of living and pattern score within the urban group of children although the effect size was small (correlation coefficient = 0.165, *P* = 0.002) and no effect in the rural group (correlation coefficient = −0.01, *P* = 0.881). Results of a *post hoc* test for interaction showed that there was a significant interaction between standard of living and urban/rural dwelling (*P* = 0.033).

### Associations between diet patterns and the child's nutritional status

Snack and fruit pattern scores were not related to the children's height but were negatively related to their adiposity as measured by BMI, subscapular skinfolds and body fat percentage (Table 6). There were no significant associations with plasma folate or vitamin B12 concentrations. In contrast, the lacto‐vegetarian pattern was not associated with adiposity, but pattern scores were positively associated with plasma folate concentrations, and negatively with vitamin B12 concentrations.

**Table 6:** Body composition and bio‐chemical measurements by quarter of pattern score*

	Snack and fruit pattern		Lacto‐vegetarian pattern	
	Q1	Q4	Q1	Q4
Height (cm)	130.8 (126.5, 134.7)	130.8 (127.6, 134.6)	**130.0 (126.2, 133.2)**	**130.4 (128.0, 135.2)** ^†^
Body mass index (kg m^−2^)	**14.9 (13.7, 16.3)**	**14.3 (13.3, 15.5)** ^†^	14.4 (13.4, 15.6)	14.4 (13.1, 15.3)
Plasma folate (nmol L^−1^)	24.1 (18.0, 32.5)	25.0 (18.7, 37.5)	**22.8 (17.2, 32.0)**	**25.2 (19.0, 35.4)** ^‡^
Plasma B12 (pmol L^−1^)	314 (249, 417)	302 (236, 350)	**332 (273, 423)**	**289 (232, 368)** ^‡^
Mid‐upper arm cincumference (cm)	18.1 (17.1, 19.8)	17.7 (16.9, 19.3)	17.7 (17.0, 19.2)	17.6 (16.5, 18.8)
Head circumference (cm)	**51.0 (50.0, 52.0)**	**50.5 (49.6, 51.4)** ^†^	50.3 (49.6, 51.3)	50.5 (49.6, 51.6)
Subscapular SF (mm)	**7.6 (6.1, 9.4)**	**7.1 (5.5, 9.3)** ^†^	7.4 (5.6, 9.4)	6.8 (5.7, 9.1)
Triceps SF (mm)	9.8 (7.8, 12.8)	9.2 (7.2, 12.1)	9.7 (7.7, 12.4)	9.1 (7.5, 11.3)
Fat weight (kg)	**7.2 (5.4, 8.9)**	**6.3 (5.0, 8.1)** ^‡^	6.8 (5.4, 8.5)	6.2 (4.9, 7.7)
Body fat %	**29.2 (23.9, 32.9)**	**25.6 (22.3, 30.5)** ^‡^	28.8 (23.5, 33.0)	25.4 (21.6, 32.2)

Q1, lowest quarter of pattern score, Q4, uppermost quarter of pattern score. SF, skinfold thickness. *Values are median (inter‐quartile range). Bold values indicate statistical significance in Wilcoxon rank‐sum test for trend across quarters of pattern score: ^†^ *P* < 0.05, ^‡^ *P* < 0.01.

## Discussion

To our knowledge, this is the first study in India to examine associations between diet patterns and demographic and body composition variables in a large number of children. We identified two main diet patterns. Variability in adherence to the diet patterns reflected biologically important differences in food intakes (Table 3). Pattern scores were predicted by the children's socio‐demographic and background characteristics, and were associated with measures of adiposity and nutritional status.

### Snack and Fruit Pattern

Many of the foods that characterised this pattern, including sweetened drinks, branded noodles and leavened breads, were shop‐bought items and few were home‐cooked foods. The demographic factors that were associated with this diet pattern were urban dwelling and being part of a nuclear family. This suggests that the pattern may have been related to a more modern style of family life. The snack and fruit pattern was also associated with being Muslim which was unexpected. It possibly reflects the fact that the Muslim religion prescribes fewer dietary restrictions than the Hindu religion. Scores for the snack and fruit pattern were inversely related to BMI and fat mass. This finding is not consistent with the increase in childhood adiposity occurring in India in conjunction with urbanisation (Misra *et al*. 2007). The snack and fruit pattern comprised healthy and unhealthy aspects, with more micronutrients from fruit but high levels of fat and sugar. Although sugar‐sweetened drinks were a discriminatory item for this pattern, intakes were low compared with typical Western populations (Harrington 2008; Hafekost *et al*. 2011). It is conceivable that if the nutrition transition continues, consumption of such drinks will increase. A recent study assessing the effect of rural to urban migration in India found that urban dwelling adults consumed up to 80% more fruit than their rural counterparts (Bowen *et al*. 2011). It is thought this is due to wider availability of produce and greater purchasing power among urban dwellers. Urban dwellers also tended to have a higher energy and fat intake.

### Lacto‐vegetarian Pattern

This pattern represents a traditional South Indian lacto‐vegetarian diet. Children adhering to this pattern were more likely to be Hindu and rural dwelling and have mothers with a low BMI. Our findings suggest that there were two groups of children with high scores on this pattern: rural children who consumed this diet because it was readily available and relatively inexpensive; higher caste Hindus who lived in the city, had a professional head of household, and consumed this diet because of tradition and religious observance. The finding that there was a weak but statistically significant association between standard of living index and lacto‐vegetarian pattern scores among children who lived in urban areas may support this suggestion. The lacto‐vegetarian pattern was also strongly positively related to plasma folate concentrations and inversely related to plasma vitamin B12. This is consistent with high vegetable consumption and low animal food intake (Joint FAO/WHO Expert Consultation on Human Vitamin and Mineral Requirements 2004). Season of FFQ administration was a predictor of pattern scores but explained a small proportion of the variance. It is possible that some of the vegetables that are characteristic of this diet are less widely available in winter months or that religious festivals are associated with changes in diet at certain times of the year.

### Associations between diet patterns and demographic characteristics

We are not aware of any data from South Asia on children's diet patterns and demographic variables. A study in urban Pakistani men of low socio‐economic status found that education and income were positively associated with ‘high meat diet’ scores and that a ‘prudent’ diet (characterised by a high intake of eggs, fish, raw vegetables and fruit) was associated with being better educated (Yakub *et al*. 2010). In addition, a large cross‐sectional study in rural parts of India, showed that low fruit and vegetable intake was associated with low socio‐economic status (Kinra *et al*. 2010). Maternal education is positively associated with adherence to a healthy diet pattern in Australia and the UK (Ambrosini *et al*. 2010; Cribb *et al*. 2011). Our data do not show such a relationship. This may be due to less variability in maternal education in this population or the fact that we did not identify a specifically ‘prudent’ or ‘healthy’ diet pattern in this cohort.

### Associations between diet patterns and child body composition

Several studies in Western populations have shown inverse associations between healthy diet patterns and measures of childhood obesity including waist circumference and overweight (Ritchie *et al*. 2007; Lioret *et al*. 2008). A study in Bengalee women aged 35 years and above found an independent positive association between adherence to a diet characterised by high intakes of hydrogenated and saturated fat, and BMI and waist circumference (Ganguli *et al*. 2011). In Pakistani men, there was a positive association between a ‘high meat’ diet and waist hip ratio (Yakub *et al*. 2010). The negative association between adiposity and the snack and fruit pattern requires explanation. It is possible that the diets of urban dwelling children in this study have only recently started to contain foods with higher fat and sugar and that the effects on body composition of these foods will become evident as the children grow older. We will be able to address this question in the continued follow‐up of these children.

### Associations between diet patterns and child folate and vitamin B12 status

Among Pakistani adults, ‘high meat’ and ‘Western’ diet patterns were associated with raised homocysteine levels (Eilat‐Adar *et al*. 2009; Yakub *et al*. 2010). We found that following a lacto‐vegetarian diet was positively associated with folate status, this is similar to a ‘high vegetable’ diet in the Pakistan study. The negative association with vitamin B12 levels may be a particular concern for the rural poor who may have limited access to animal foods. The lack of association between these nutrient biomarkers and the snack and fruit pattern might be expected as variations in consumption of many of the foods that characterised this pattern would not contribute to significant differences in intakes of these nutrients.

### Strengths and limitations

We used an FFQ that was developed for the study. The retained patterns together explained 16.6% of the variation in the 52 food groups. The proportion of variance explained is dependent on the number of variables entered into a PCA and the number of components retained (Newby & Tucker 2004; Crozier *et al*. 2006). This makes it difficult to compare diet pattern studies directly. However, in other diet patterns analyses of European data, where similar numbers of input variables have been used a comparable proportion of variance was explained by two patterns (Crozier *et al*. 2006; Robinson *et al*. 2007; Fisk *et al*. 2011). Although FFQs may be subject to measurement error, patterns defined using FFQ data have been shown to be comparable to patterns defined using other assessment methods, and pattern scores from different methods are highly correlated (Hu 2002). Furthermore, a number of studies have shown associations between diet patterns and chronic disease outcomes (Fung *et al*. 2001; Osler *et al*. 2001; Miller *et al*. 2010), highlighting patterns analysis as a potentially useful technique to gain a greater understanding of the importance of diet behaviour.

The multivariate models (Table 5) explained less of the variance in snack and fruit pattern scores compared with the lacto‐vegetarian pattern. This may be due to variability in adherence to the snack and fruit pattern being associated with unmeasured factors such as family income, taste preferences, marketing and social desirability.

A strength of our data is that we assessed folate and vitamin B12 status. The association between folate and vitamin B12 concentrations and the lacto‐vegetarian pattern scores was as expected, and adds strength to the validity of our dietary data. It is conceivable that activity could have been associated with both adiposity and the children's dietary patterns. We were not able to assess such relationships at this age. However, data collected when the children were younger (mean, SD age 7.8, 1.1 years) indicated that the children were relatively inactive and there was little variability in activity between children (Kehoe *et al*. 2012).

The absence of an obviously ‘healthy’ or ‘prudent’ pattern in our analysis is of interest and is in contrast to many analyses conducted in the United States and Europe (Newby & Tucker 2004). As with many countries, it has been observed that over recent years, there is increasing prevalence of advertising for high sugar and high energy foods aimed at children in India (de Cruz 2004; Kaur & Singh 2006; Consumers International 2008). It will be important to determine how diet patterns are affected by such influences as a result of the nutrition transition.

In conclusion, we have shown that meaningful diet patterns can be identified in this population and these patterns are associated with demographic and body composition variables in Indian children. These findings may be helpful in developing food‐based dietary guidelines in this population and also in designing interventions aimed at prevention of overweight and other risk factors for chronic disease. As we follow this cohort, we will use these patterns to study associations between diet and risk factors for chronic disease in childhood and later life.

## Source of funding

This work was funded by the Parthenon Trust, Wellcome Trust and the Medical Research Council.

## Conflicts of interest

The authors declare that they have no conflicts of interest.

## Contributions

CHDF designed the study, SMR and BMM provided significant advice, GVK and SRV supervised the data collection, AMG conducted the statistical analysis, SHK wrote the manuscript. All authors contributed to the interpretation of the results and were involved in preparing the final manuscript.

## Supporting information

Appendix S1: Mysore Cohort Food Frequency Questionnaire.
